# Factors Associated With Social Isolation Among Older Adults: The Singapore Chinese Health Study

**DOI:** 10.1093/geroni/igaa057.543

**Published:** 2020-12-16

**Authors:** Jon Barrenetxea, Yang Yi, Woon Puay Koh, Feng Qiushi

**Affiliations:** 1 Duke-Nus Medical School, Singapore, Singapore; 2 National University of Singapore, Singapore, Singapore

## Abstract

Social isolation is a determinant of mortality and well-being among older people. Factors associated with isolation could be different in societies where older adults live mainly with family, as individuals might feel isolated despite living with others. We studied the factors associated with isolation among 16,948 older adults from follow-up 3 of the Singapore Chinese Health Study, a population-based cohort of older Singapore Chinese (mean age of 73, range: 61-96 years). We defined social isolation as having “zero hour per week” of participation in social activities involving 3 or more people and scoring the lowest decile on the Duke Social Support Scale of perceived social support. We used multivariable logistic regressions to compute odds ratio (OR) and 95% confidence interval (CI) for factors associated with likelihood of social isolation. Although only 14.4% of isolated participants lived alone, living alone remained a significant factor associated with isolation (OR 1.93, 95% CI 1.58-2.35), together with cognitive impairment (OR 1.73, 95% CI 1.46-2.04) and depression (OR 2.44, 95% CI 2.12-2.80). Higher education level was inversely associated with isolation (p for trend<0.001). In stratified analysis, among those living alone, compared to women, men had higher odds of social isolation (OR 2.18, 95% CI 1.43-3.32) than among those not living alone (OR 0.99, 95% CI 0.84-1.17) (p for interaction<0.001). Our results showed that living alone, cognitive impairment and depression were indicators of isolation among older Singaporeans. In addition, among those living alone, men were more likely to experience social isolation than women.

